# Effect of an “Autogenous Leukocyte Platelet-Rich Fibrin Tooth Graft” Combination around Immediately Placed Implants in Periodontally Compromised Sites: A Randomized Clinical Trial

**DOI:** 10.1155/2022/4951455

**Published:** 2022-02-22

**Authors:** Walid ElAmrousy, Dalia Rasheed Issa

**Affiliations:** Department of Oral Medicine and Periodontology, Faculty of Oral and Dental Medicine, Kafrelsheikh University, Kafr El-sheikh, Egypt

## Abstract

**Objective:**

Autogenous tooth bone graft (ATBG) was suggested as a source for bone grafting materials, especially as they have similar chemical composition to bone. This study goal was to assess the clinical and radiographic consequences of ATBG with or without L-PRF on bone deposition around immediate implants placed in periodontally hopeless sites.

**Materials and Methods:**

26 patients, with periodontally diseased teeth, underwent random assignment to receive the surgical protocol either with L-PRF over ATBG around immediately inserted implants (test group) or without it (control group). Clinical examination was observed. Radiographically, bone changes horizontally and vertically to determine marginal bone loss (MBL) and mesiodistal bone changes were made at the base line and 6 and 9 months after implant insertion. Statistical analysis utilizing paired Student's *t*-test was used for comparing results within the same group, whereas an independent-sample *t*-test was used for intergroup variable comparison.

**Results:**

All implants met the criteria of success without any complications at the follow-up period. Nonsignificant differences were detected between horizontal bone alterations in both groups at 6 and 9 months (*P* > .001). The test group showed statistically significant lower MBL than the control group (*P* < .001). The mesiodistal bone gain in the test group was significantly higher than that of the control group at the 6-month period (*P* < .001). The mesiodistal bone loss in the control group was significantly higher than that of the test group at the 9-month period (*P* < .001).

**Conclusion:**

The ATBG- L-PRF combination therapy enhances new bone formation and appeared to be a favorable procedure with immediate implant placement, particularly in severe periodontitis cases.

## 1. Introduction

Periodontitis is an inflammatory condition that can result in the creation of periodontal pockets, attachment loss, gingival recession, and tooth loss. Periodontitis affects roughly 10%–15% of the global population. Periodontal disease development is marked by alveolar bone resorption, which is a predictor of tooth loss [1].

Nowadays, immediate implants placed into freshly extracted sockets are gaining popularity since they require few surgical procedures and preserve bone architectures, thus saving intervention time and enhancing patients' satisfactions. Furthermore, the immediate implants survival rate is comparable to that of the delayed implants in healed bone [2].

However, immediate implantation in the molar area frequently poses a lack of bone amount in the newly extracted socket due to bulky roots. Guided bone regeneration (GBR) is commonly used to augment the defected bone surrounding the implant [3].

Because of their osteogenic, osteoinductive, and osteoconductive characteristics, autogenous bone grafts are considered as the gold standard for bone augmentation techniques. On the other hand, autogenous bone grafts have a number of drawbacks, including the need of a second surgical intervention, production of a small quantity of bone, donor site morbidity, postoperative pain, patient discomfort, and possible neurological injury at the donor site [4]. Allogenic and xenogenic bone substitute materials, on contrary, have minimal osteogenic impact, are expensive to treat, not accepted by patients, and have high susceptibility to infection and immunological reactions [5].

In 2008, ATBG was introduced and used for the first time as a bone grafting material for GBR [6]. The tooth content is extremely similar to that of the alveolar bone. The enamel inorganic, organic, and water content is 95 percent, 0.6 percent, and 4 percent, respectively. However, in the dentin, the percentages are 70 to 75 percent, 20 percent, and 10 percent, respectively. They are 65 percent, 25 percent, and 10 percent, respectively, when compared to the alveolar bone content [7].

Calcium phosphates such as hydroxyapatite, amorphous calcium phosphate, tricalcium phosphate, and octacalcium phosphate make up the majority of the inorganic composition of teeth. Noncollagenous proteins found in the dentine organic matrix such as osteocalcin, sialoprotein, osteonectin, and phosphoprotein are responsible for bone calcification, as well as growth factors such as bone morphogenetic proteins, LIM mineralization protein 1, and insulin-like growth factors. Consequently, ATBG has osteoinductive and osteoconductive characteristics similar to autogenous bone, making it the ideal material for bone grafting [8].

ATBG derived from extracted dentition was recently tested in bone deformities and found to be effective. Furthermore, patient acceptance to ATBG is high, especially when applied with immediately placed implants [9].

Demineralization increases the osteoinductive potential by exposing organic materials of teeth to the surface, increasing porosity and surface area, and reducing crystalline form [10]. The biocompatibility and osteoinductivity of the demineralized dentin matrix (DDM) are comparable to those of the demineralized bone matrix. Undemineralized dentin (UDD) showed high bone regenerative ability. After tooth sterilisation and cleansing, UDD can be easily retrieved from a dentine-grinding machine. Only Korea tooth bank has partially demineralized dentin matrix (PDDM) that could be obtained by partial dentin demineralization [11].

In rabbit calvarial bony deformities, Kizilda et al. investigated the effects of ATBG in conjunction with PRF on new bone formation. ATBG coupled with PRF significantly promoted new bone formation and improved bone repair [12]. Kizilda et al. reported the effects of PRF combined with ATBG on rabbit peri-implant bony defects in 2020. In rabbits with peri-implant bone deficiencies, they found that combining ATBG with PRF resulted in increased new bone formation and improved bone/implant contact [13].

L-PRF (leukocyte and platelet-rich fibrin) is the second generation of platelet concentrate, created simply by centrifuging blood at 2700 rpm for 12 min, which is easy to prepare and does not involve biochemical blood treatment [14]. Increased release of transforming growth factor beta-1 (TGF beta-1), platelet-derived growth factor AB (PDGF-AB), and vascular endothelial growth factor (VEGF) levels are induced by L-PRF. Moreover, L-PRF has been shown to improve wound healing in both soft and hard tissues [15]. Numerous studies reported the L-PRF potential for bone and soft-tissue regeneration, without inflammatory reactions, which can be used alone or in combination with graft substitutes. This stimulates haemostasis, angiogenesis, and bone regeneration [16, 17].

The fundamental hypothesis of this study was that combining PRF with ATBG after immediate implant insertion would boost graft particle stabilization and new bone formation in less time. Therefore, the goal of this study was to assess, clinically and radiographically, the use of ATBG with and without L-PRF around immediately inserted dental implants in periodontally impaired teeth. To the knowledge of the authors, no study to date has addressed the effect of ATBG combined with L-PRF around immediate implant placement.

## 2. Methods and Materials

### 2.1. Participant Selection

It was a prospective, randomized, and blinded clinical trial in which a total of 26 patients, aged from 18–50 years, were chosen from the Periodontology and Oral Medicine Department of the Faculty of Oral and Dental Medicine, Kafrelsheikh University. All participants involved in this study signed written informed consent. From January 2020 to April 2021, this study was undertaken at the Periodontology and Oral Medicine Department, Faculty of Oral and Dental Medicine, Kafrelsheikh University, Egypt. The Research Ethics Committees of Kafrelsheikh University had approved the study (KD/03/20). This clinical trial was registered under a clinical trial registration number (NCT04795102).

Sample size calculation was undertaken via G power version 3.1 statistical software based on the following preestablished parameters: an alpha-type error of 0.05, a power test of 0.80, and a total sample of 26 subjects (13 subjects for each group), which appeared to be sufficient.

The criteria for inclusion were (1) teeth with buccal bone destruction by periodontal disease and needing extraction (horizontal or vertical bone defect); (2) absence of acute inflammation; (3) absence of uncontrolled systemic illness that would preclude implantation; (4) good dental and systemic healthy conditions; and (5) patients willing and able to return for multiple follow‐up visits.

Patients with systemic illnesses, psychological abnormalities, parafunctional habit, smokers or alcoholics, pregnant and lactating patients, patients undergoing or recently completed radiotherapy or chemotherapy, patients on drugs affecting the healing process, or patients with endodontically treated teeth were excluded from the present study.

### 2.2. Patient Grouping

Patients were randomized into a test group where L-PRF was used over ATBG around immediate implants placed after tooth extraction or a control group where ATBG was used without L-PRF. Randomization was carried out by a means of computer-generated software (Random Allocation Software, Version 1.0, May 2004), and the treatment code for each patient was designated into a numbered, opaque, sealed envelope that was unwrapped prior to each surgery.

### 2.3. Preoperative Work-Up

Presurgically, all participants had a thorough intraoral examination and CBCT analysis using a specialized unit (Scanora 3D, Soredex Oy, Tuusula, Finland) to examine the affected teeth to be extracted as well as the surrounding bone condition.

Two weeks prior to surgery, all participants received professional oral hygiene instructions in conjunction with scaling and root planning. Preoperative antibiotics (Augmentin, 1 g, SmithKline Beecham Pharmaceuticals; amoxicillin clavulanic acid 1 g every 12 hours) were administered 3 days prior to surgery, and 0.2% chlorhexidine mouthwash was administered 1 week prior surgery to prevent infection and improve plaque control [18].

Precise baseline measurements of the bone width, the depth, and the bone level using CBCT were recorded for proper implant selection and planning for implant placement.

### 2.4. Autogenous Tooth Bone Graft Preparation

**Figure 1:**
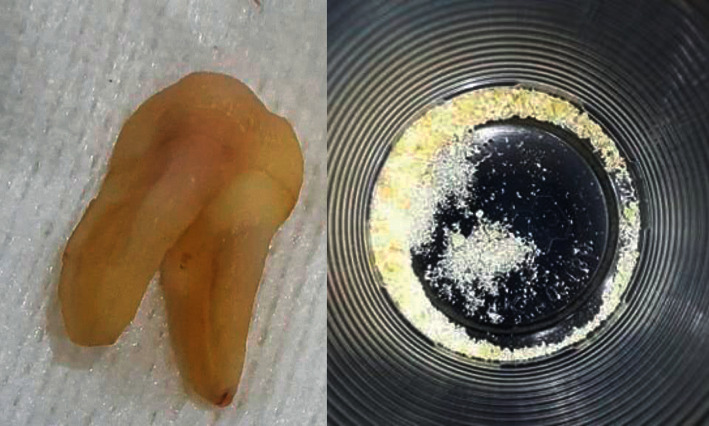
Preparation of ATBG material was performed from the extracted tooth.

For all cases, the preparation of ATBG material was performed by the same operator. ATBG was produced using a vacuum ultrasonic autoclaved bone preparation machine (VacuaSonic®, Korea) and was derived from teeth that needed to be extracted (Figure 1). Thirty minutes presurgically, autologous teeth were removed using a traumatic tooth extraction under Primacaine® (4 percent Articaine, 1/100000 adrenaline, ACTEON) local anaesthetic. Using fissure or flame tungsten carbide burs, caries, restorations, diseased dentine, remaining periodontal ligaments, and/or dental biofilm were removed. The teeth were then cleaned using sterile physiological saline before being properly air-dried.

Teeth fragments were ground down in the crushing chamber for three minutes to 300- and 1200-micron particles. Particles with a diameter of less than 300 microns were eliminated. The resulting particles were soaked in a sterilised glassy container containing 0.5 molar NaOH and 20% ethanol for 10 minutes to dissolve any organic residues, germs, and poisons discovered in dentine. Finally, they were washed in a phosphate-buffered sterile saline solution [19].

### 2.5. L-PRF Preparation

Choukroun et al.'s technique [14] was used to prepare the L-PRF clot 20 minutes prior to surgical intervention. A total of 20 milliliters of blood was extracted from the antecubital vein and placed in two sterile vacuum tubes, and then, centrifugation for 12 minutes at 400 g relative centrifugation force with 2800 rpm was performed using Daiki DT-4000 Centrifuge^TM^ (IonlabEquipamentosLaboratoriais e HospitalaresLtda, Brazil). Using sterilised metal plates, L-PRF clots were identified and gently squeezed.

### 2.6. Surgical and Prosthetic Procedures

**Figure 2:**
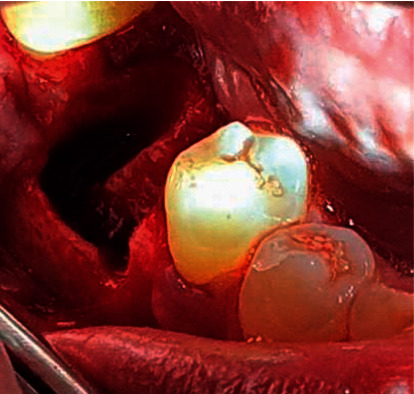
Atraumatic extraction and full-thickness flap were performed.

**Figure 3:**
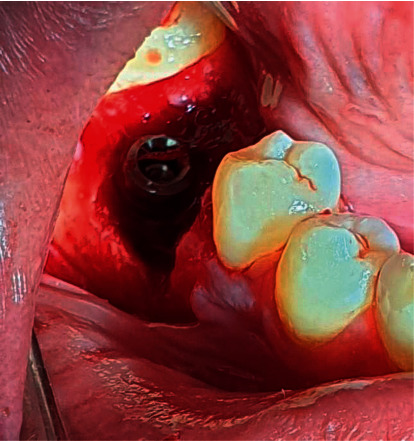
The implant fixture was placed 3-4 mm below the labial gingival margin.

**Figure 4:**
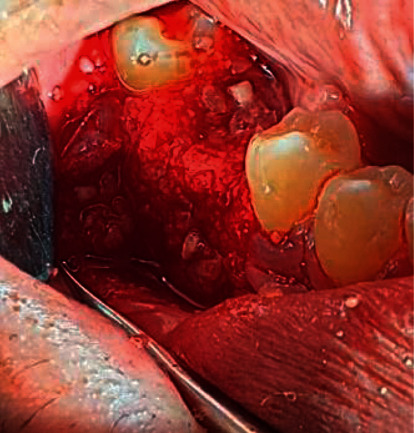
ATBG was used to augment the defective bone and the gaps between the socket boundaries and the implanted fixture.

**Figure 5:**
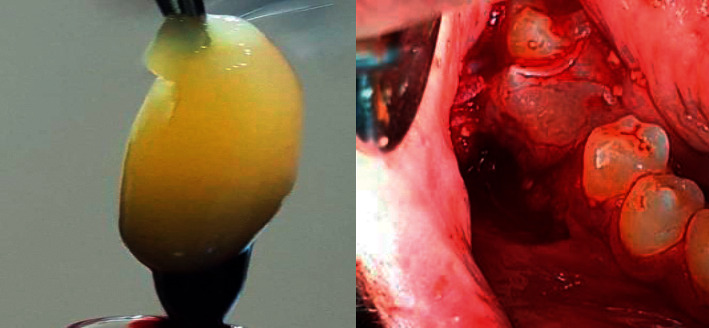
L-PRF was used to cover the graft materials.

All surgical interventions were operated by one surgeon. Extraorally, 2% chlorhexidine and intraorally 0.2% chlorhexidine were used as the antiseptic regimen. Then, the tooth was atraumatically extracted under a local anaesthetic agent using a periotome and forceps. Full-thickness crestal with distal vertically released incisions were performed (Figure 2). Buccal and lingual flaps were reflected to reveal the extraction socket and bone deficiency labially. Before implant bed drilling, inflammatory granulation tissues were eliminated with forceful curettage and copious irrigation using 0.9% saline solution. Subsequently, the implant bed was drilled 3–5 mm below the socket base and centered mesiodistally. Then, the implant fixture was placed 3-4 mm below the labial gingival margin (Figure 3). In the control group, ATBG was used to augment the defective bone and the gaps between the socket boundaries and the implanted fixture (Figure 4). The same was performed in the test group, but L-PRF was used to cover the graft materials (Figure 5). Finally, the flaps were repositioned and sutured.

### 2.7. Postoperative Care

To avoid wound infection, amoxicillin clavulanic acid 1 g every 12 hours and analgesics were prescribed postoperatively. The final titanium abutment and zirconia prosthesis were positioned after 6 months of surgery. CBCT scans were performed 6 and 9 months postsurgically.

### 2.8. Outcome Measurements

#### 2.8.1. Implant Success

According to Papaspyridakos et al. [20] and Karthik et al. [21], the implant success criteria were the absence of mobility, peri-implant infections, or peri-implant radiolucencent lesions, pocket probing depth (PPD) less than 5 mm, and vertical bone resorption less than 1.5 mm in the first year.

### 2.9. Clinical Assessment

Swelling, numbness, and wound dehiscence were checked at the 3^rd^ and 7^th^ day postoperatively.

### 2.10. Radiographic Assessment

**Figure 6:**
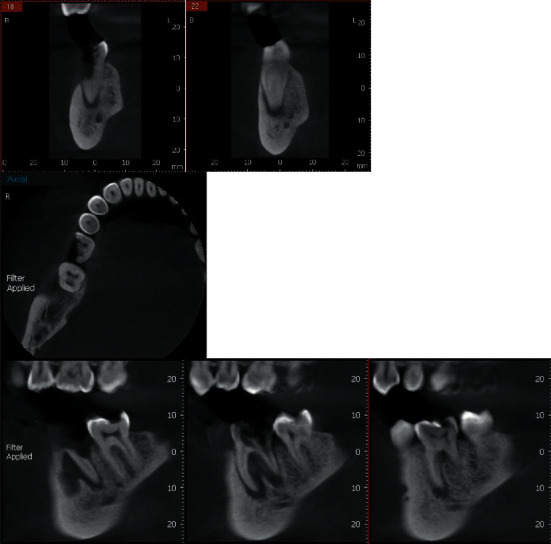
CBCT scanning preoperatively.

**Figure 7:**
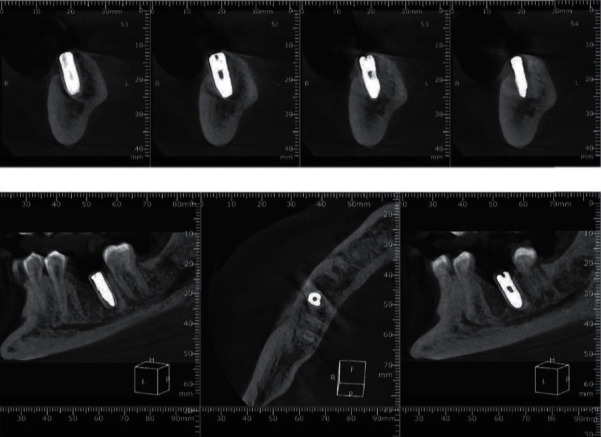
CBCT scanning postoperatively.

All patients were measured by CBCT scanning preoperatively and 6 and 9 months postsurgically (Figures 6 and 7). All measurements were performed by the same calibrated masked operator using a specific anatomic landmarks in the same location at baseline and throughout the observation period. Buccolingual ridge width measurements were calibrated in the level of 2 mm from the implant margin. Considering the vertical bone changes, marginal bone loss (MBL) was measured by drawing two lines buccally and lingually from the inferior alveolar canal to the alveolar crest. Using the cementoenamel junction of the adjacent teeth, a reference line was created, and then, a measurement from that line was made to detect the mesiodistal bone changes. The implant's central line was considered as a reference line vertically in the CBCT radiograph, and the whole recorded points were at right angle to that central line.

### 2.11. Statistical Analysis

The participants' demographic data, personal habits, and the tooth extraction results were arranged by ExcelTM software (Microsoft, USA) and exported to be analysed statistically by SPSS v21.0 TM software (SPSS Inc., USA). A paired *t*-test was performed for comparing changes in alveolar width and height and statistical significance when *P* < 0.001. Student's T- and chi-square tests were used for intergroup comparisons, with *P* value < 0.001 representing statistical significances. An independent-sample *t*-test with the SPSS was used for comparing different variable gains at different follow-up intervals with statistically significant outcomes when *P* values < 0.001.

## 3. Results

The total of 26 subjects (12 males and 14 females), with 18–50 years (mean 35.8 ± 8.6 years) age range, were included in this study from January 2020 to March 2021. Of these patients, 13 belonged to the group of ATBG covered with L-PRF clots around immediate implants in freshly extracted sockets (group A) or ATBG alone around immediate implants in freshly extracted sockets (group B). All implants were inserted in the posterior mandibular region.

### 3.1. Implant Success

All the implants in both groups reached the success criteria during the entire observation period. Neither the implants nor the graft materials had any biological and mechanical complications, such as peri-implantitis and infection during the follow-up period.

### 3.2. Clinical Assessment

There were different levels of pain and swelling in both groups at day 7 after surgery. However, All treated sites healed uneventfully, and all 26 patients had no postoperative complications.

### 3.3. Radiographic Assessment

Dimensional alterations that occurred during healing for all sites are reported in Tables 1 and 2.

### 3.4. Horizontal Dimensional Changes

**Figure 8:**
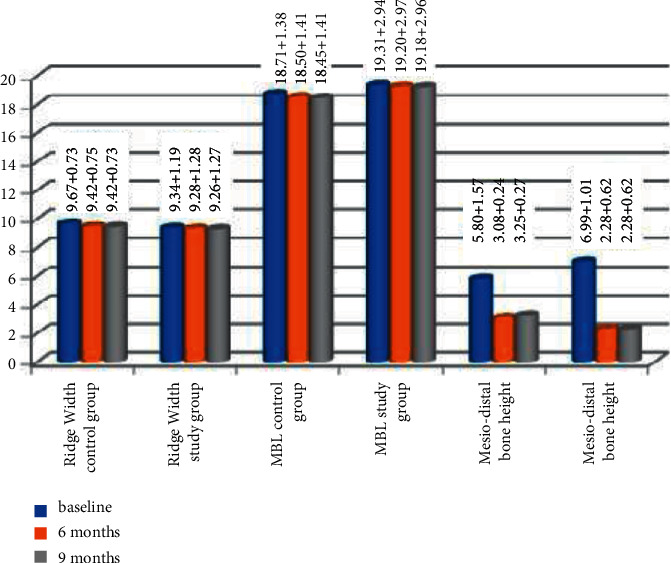
Bar chart representing the mean and standard deviation of radiographic measurement in the two studied groups in different observation periods.

**Figure 9:**
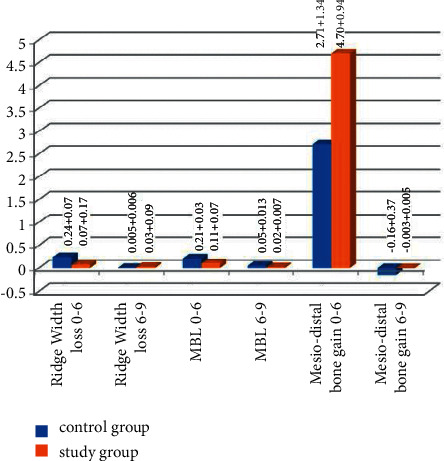
Bar chart representing the comparison of the mean and standard deviation of radiographic measurement between the two studied groups in different observation periods.

The width of the alveolar ridge was measured at the level of 2 mm apical to the implant margin. The control group demonstrated a statistical significant reduction in the ridge width at 6 and 9 months (9.42 + 0.75 mm and 9.42 + 0.73 mm), respectively, compared to the baseline (9.67 + 0.73 mm) (*P* < 0.001). At 6 and 9 months, the ridge width decreased in the test group (9.28 + 1.28 mm and 9.26 + 1.27 mm), respectively, from the baseline (9.34 + 1.19 mm) with no statistically significant difference (*P* > 0.001). The horizontal bone changes in the control group at 6 and 9 months were 0.24 + 0.07 mm and 0.005 + 0.006 mm and in the test group at 6 and 9 months were 0.07 + 0.17 mm and 0.005 + 0.006 mm. No significant difference was detected between 6‐ and 9‐month observation periods in the two groups ((*P* > 0.001); Figure 8). No statistically significant difference was found between mean ridge width loss in both groups at 6 and 9 months ((*P* > 0.001); Figure 9).

### 3.5. Vertical (Labiolingual) Changes

#### 3.5.1. MBL

MBL at 6 and 9 months was 0.21 + 0.03 mm and 0.05 + 0.013 mm in the control group and 0.11 + 0.07 mm and 0.02 + 0.007 mm in the test group, respectively. Both groups showed statistically significant MBL at 6 and 9 months compared with initial values (*P* < 0.001). There was a significant difference between 6- and 9-month observation intervals in the two groups ((*P* < 0.001); Figure 8).

Throughout all observation periods, the control group showed statistically significant MBL than the test group ((*P* < 0.001); Figure 9).

### 3.6. Mesiodistal Bone Gain

The mesiodistal bone gain at 6 months was (2.71 + 1.34) mm and (4.70 + 0.94) mm in the control group and the test group, respectively. Also, at 9 months, it was (−0.16 + 0.37) mm and (−0.003 + 0.005) mm in the control group and the test group, respectively. At 6 and 9 months, both groups revealed a statistically significant difference compared with the initial value (*P* < .001), while no significant differences were observed between 6- and 9-month follow-up periods in both groups (*P* > .001). The mesiodistal bone gain in the test group was significantly higher than that of the control group at the 6-month period (*P* < .001; Figure 8). On the other hand, the mesiodistal bone loss in the control group was significantly higher than that of the test group at the 9-month period (*P* < .001; Figure 9).

## 4. Discussion

Immediate implant placement in freshly extracted sockets helps to preserve peri-implant hard and soft tissues, enhances the implants` survivals, reduces the treatment duration, and keeps patients satisfied [22].

Several biomaterials were used for alveolar ridge preservation to serve as a space-creating scaffold and to contribute to the biological healing process [23]. The extracted teeth reveal structural and biological capacity to improve bony defect reconstruction due to its common embryological origin and similar composition [24]. ATBG can overcome many limitations associated with other grafting materials such as lower morbidity and lower resorption rate compared to bone autografts, eliminating the possibility of cross infection, immune reactions, and differences in donor health conditions and processing procedures associated with allografts and xenografts [25]. Furthermore, the presence of platelets and leukocytes in L-PRF, which release growth factors and cytokines, has been found to boost the healing process and stimulate tissue regeneration. The use of L-PRF as a membrane over ATBG promotes angiogenesis and new bone deposition by allowing stem cells and osteogenic cells to migrate through their fibrin mesh and the gradual release of growth factors [26].

In the present study, the efficacy of ATBG and L-PRF was assessed that could stimulate new bone formation in the immediate implant placement.

There was a good tissue acceptance with no postoperative complications. This can be attributed to the absence of antibody production against ATBG [27]. Additionally, removal of all old restorations, pulp, cementum, and bacteria by using the demineralization and sterilisation process reduces the inflammatory mediator secretion.

Findings from the present study showed a ridge width reduction in the test group at 6 and 9 months compared to the baseline with no statistically significant difference. This could be due to the loss of the bundle bone, where the periodontal ligament fibers invest, following tooth extraction [28]. This can be also in accordance with many systematic reviews which claimed that no substitute material was able to completely preserve the alveolar ridge after tooth extraction, but may limit buccal plate resorption to a certain extent [29, 30]. The two groups reported no statistically significant difference between mean ridge width loss at 6 and 9 months. This finding may be explained by that placement of ATBG in the void between the implant and the walls of the fresh extraction socket in both groups slightly counteracted the contraction of the buccal hard-tissue plate that normally occurs during healing.

Interestingly, further analysis of MBL and mesiodistal bone gain was detected during the present study. At 6 and 9 months, the mean MBL in the test group was less than that of the control group. On the other hand, at 9 months, the mean mesiodistal bone gain in the test group was markedly improved than that of the control group. After loading, there was a minimal mesiodistal bone loss which was higher in the control group than that of the test group. This indicates that the addition of L-PRF to ATBG has been recommended as an alternative approach to increase bone formation, enhance implant stability, favor osseointegration, and accelerate tissue maturation and healing [31]. Similarly, this could be related to results obtained by Kizildag et al. [12] who reported that there was a significant increase in BMP-2 level by using PRF with ATBG which increased the stabilization of graft particles. Furthermore, Pichotano et al. [32] reported that the addition of L-PRF into the maxillary sinus resulted in increased amount of newly formed bone and accelerated bone graft maturation, allowing early implant placement after sinus augmentation. Additionally, in a case report by Andrade et al. [26], it was found that the incorporation of L-PRF effect with ATBG appeared to maximize the regenerative process and the amount of newly formed bone was significantly increased.

Some unexplored variables can have a significant influence on the oral environment. The use of probiotics [33] and natural compounds can modify clinical and microbiological parameters in periodontal patients [34]. Moreover, statins and other biomaterials [35, 36] could have an effect also in the response to the technique described in the present report. All these variables should be considered in future clinical trials.

In the present study, there are limitations which should be taken into consideration. These include small sample size, short follow-up period, the need for second reentry surgery, and postoperative histological assessment to confirm the quality and quantity of newly formed bone and the healing nature. However, to the best of our knowledge, this is the first study to compare clinical and radiographic outcomes of the effect of ATBG combined with L-PRF around immediate implant placement. Further studies with a larger sample size and long-term observations would correspond with the findings presented here.

## 5. Conclusions

Within the limits of the present study, our data demonstrated that the addition of L-PRF to the ATBG increased the newly formed bone and influenced the healing process and regeneration in the immediate implant placement. Collectively, ATBG- L-PRF combination therapy appeared to be a favorable procedure with immediate implant placement, particularly in severe periodontitis cases.

## Figures and Tables

**Table 1:** Radiographic measurement of the two studied groups in different observation periods.

Parameter	Control group mean + SD	*P*	Study group mean + SD	*P*
Ridge width baseline 6 months	9.67 + 0.73, 9.42 + 0.75	<0.001^*∗*^	9.34 + 1.19, 9.28 + 1.28	>0.001 (NS)
Ridge width 6 months 9 months	9.42 + 0.75, 9.42 + 0.73	>0.001 (NS)	9.28 + 1.28, 9.26 + 1.27	>0.001 (NS)
MBL baseline 6 months	18.71 + 1.38, 18.50 + 1.41	<0.001^*∗*^	19.31 + 2.94, 19.20 + 2.97	<0.001^*∗*^
MBL 6 months 9 months	18.50 + 1.41, 18.45 + 1.41	<0.001^*∗*^	19.20 + 2.97, 19.18 + 2.96	<0.001^*∗*^
Mesiodistal bone height baseline 6 months	5.80 + 1.57, 3.08 + 0.24	<0.001^*∗*^	6.99 + 1.01, 2.28 + 0.62	<0.001^*∗*^
Mesiodistal bone height 6 months 9 months	3.08 + 0.24, 3.25 + 0.27	>0.001 (NS)	2.28 + 0.62, 2.28 + 0.62	>0.001 (NS)

**Table 2:** Comparison between the two studied groups for osseous measurement in different observation periods.

Parameter	Control group mean + SD	Study group mean + SD	*P*
Ridge width loss 0−6 months	0.24 + 0.07	0.07 + 0.17	>0.001 (NS)
Ridge width loss 6−9 months	0.005 + 0.006	0.03 + 0.09	>0.001 (NS)
MBL 0–6 months	0.21 + 0.03	0.11 + 0.07	<0.001^*∗*^
MBL 6–9 months	0.05 + 0.013	0.02 + 0.007	<0.001^*∗*^
Mesiodistal bone gain 0–6 months	2.71 + 1.34	4.70 + 0.94	<0.001^*∗*^
Mesiodistal bone gain 6–9 months	−0.16 + 0.37	−0.003 + 0.005	<0.001^*∗*^

## Data Availability

The data are available upon request from the corresponding author.
